# Prescription for asthma

**DOI:** 10.4103/0970-2113.71973

**Published:** 2010

**Authors:** Vishal Sharma, Sourabh Aggarwal, Alka Sharma

**Affiliations:** *Department of Medicine, University College of Medical Sciences, New Delhi, India. E-mail: drsourabh79@gmail.com*

Sir,

We read the article ‘Prescription for asthma’[1] by Pandey A *et al*. with interest. We applaud the authors for conducting such a study in Indian setup and would like to share our views on the same.

Since the study was done on patients coming to hospital for treatment, the authors are not fully correct in saying that the ‘prevalence’ of asthma is higher in males. Prior studies have shown that female sex is associated with an increased risk of asthma.[2] Also, the authors did not mention whether the source of data was from government hospitals or private setups, since financial constraint could have significant effect on prescription of multi-drugs versus single drug for the treatment of asthma. Nonetheless, the authors have done a commendable job in view of asthma patients and their efforts need to be appreciated.

## References

[1] Pandey A, Tripathi P, Pandey RD (2010). Prescription pattern in asthma therapy at Gorakhpur hospitals. Lung India.

[2] Jindal SK (2006). Respiratory disease epidemiology in India. Lung India.

